# Risk of Asymptomatic Bacteriuria among People with Sickle Cell Disease in Accra, Ghana

**DOI:** 10.3390/diseases5010004

**Published:** 2017-02-15

**Authors:** Eric S. Donkor, Jonathan A. Osei, Isaac Anim-Baidoo, Samuel Darkwah

**Affiliations:** 1 Department of Medical Microbiology, School of Biomedical and Allied Health Sciences, University of Ghana, Accra, Ghana; 2 Department of Medical Laboratory Science, School of Biomedical and Allied Health Sciences, University of Ghana, Accra, Ghana; Pilolosei@yahoo.com (J.A.O.); anim-baidoo@chs.edu.gh (I.A.-B.); kwekuadarkwah@gmail.com (S.D.)

**Keywords:** bacteriuria, urine, *Staphylococcus*, sickle cell disease

## Abstract

Asymptomatic bacteriuria (ASB) is benign except in certain medical conditions such as pregnancy and immunosuppression. In Ghana, there are hardly any studies on urinary infections among sickle cell disease (SCD) patients, and the few studies carried out in Africa focused on pediatric SCD populations. The current study aimed to investigate the risk of ASB among SCD patients at a tertiary hospital in Ghana. This was a cross-sectional study involving 110 SCD patients and 110 age and sex matched healthy controls. Urine specimens were collected from all the study subjects and analyzed by standard microbiological methods. Demographic information were also collected from the study subjects. The overall ASB prevalence was significantly higher among SCD patients (17.2%) than among the control group (8.2%), and the relative risk was 2.11 (*p* = 0.0431; CI = 1.00–4.45). Being female was as a predictor of ASB among the SCD patients (OR = 14.76; CI = 11.23–18.29; *p* = 0.0103). The most common organism isolated from the study participants was coagulase negative *Staphylococcus* species (4.1%), followed by *Escherichia coli* (2.7%); etiology of ASB in the SCD patients was more diverse compared to healthy people. All the *E. coli* isolates were susceptible to amikacin, sparfloxacin and norfloxacin but resistant to ampicillin.

## 1. Introduction

Urine samples are normally sterile and the presence of bacteria in urine defines bacteriuria, which is considered significant if the bacterial count is greater than 1 × 10^5^ per mL [1,2]. Significant bacteriuria may be either symptomatic or asymptomatic. The former defines urinary tract infection and is associated with symptoms such as dysuria, pyuria, and frequent urination [2]. In asymptomatic bacteriuria (ASB), the accompanying symptoms of urinary tract infection are absent, and some of the associated risk groups of ASB include elderly people, and patients with diabetes, bladder catheters and sickle cell disease (SCD) [3,4,5]. The prognostic significance of ASB is based on the fact that it could lead to an increased risk of pyelonephritis and renal impairment in certain medical conditions such as diabetes mellitus and pregnancy [6,7]. Several bacterial organisms have been implicated in bacteriuria, but the common ones include *Escherichia coli* and *Staphylococcus* spp. [2,3]. In ASB, alterations in host-pathogen interaction may be responsible for the absence of symptoms despite the presence of urinary pathogens [8].

Sickle cell disease (SCD) refers to a number of genetic disorders associated with structurally abnormal hemoglobin resulting in the episodic formation of sickle-shaped red blood cells and several clinical manifestations. The underlying genetic abnormality is a point mutation (GTG for GAG) in the gene for β-globin on chromosome 11, leading to the replacement of a glutamic acid residue with valine on the surface of the protein (termed HbS) [9]. About 75% of people with SCD live in sub-Saharan Africa, where many infectious diseases also take their toll [10]. As a result of impairment of their immune system, SCD patients are relatively more prone to infections, especially with encapsulated bacteria. Evidence from several studies indicates that SCD patients have a relatively higher risk of developing bacteriuria compared to their healthy counterparts [11,12]. Urinary infections in people with SCD may result in long-term renal dysfunction [13], which could be partly forestalled through early detection and treatment of the infections. In Ghana, so far no study has investigated urinary infections among SCD patients, and the few studies carried out in Africa have focused on pediatric SCD populations. The current study aimed to investigate the risk of ASB among SCD patients at a tertiary hospital in Ghana.

## 2. Methods

### 2.1. Study Site, Design, and Sampling

The study was conducted at the Korle-Bu Teaching Hospital (KBTH) in Accra, the capital city of Ghana. The population of Ghana is about 25 million [14], and the prevalence of sickle cell disease is 1.9% of all births per year [15]. The out-patient morbidity reports compiled by the Ghana Health Service in 2002 and 2003 ranked SCD at positions 37th and 36th, respectively [16]. There are over 100 hospitals in Ghana and a national health insurance scheme has been in operation since 2004. KBTH is the largest hospital in Ghana with a bed capacity of about 2000 and 17 clinical and diagnostic departments including a Sickle Cell Centre [17]. The hospital has an average daily attendance of 1500 patients and about 250 patient admissions [17]. Generally, penicillin prophylaxis is administered to SCD children less than five years old in Ghana [18].

Using a 95% confidence level, 4% estimated asymptomatic bacteriuria prevalence reported previously among SCD patients [18] and 5% allowable error, 110 consecutive SCD outpatients visiting the Sickle Cell Clinic of KBTH for review were recruited in the study. We excluded SCD patients who had symptoms of urinary tract infections and those who had taken antibiotics two weeks or earlier prior to the study. A control group comprising 110 age-matched and sex-matched subjects, without SCD were recruited randomly from the environs of KBTH. The exclusion criteria in the recruitment of the controls were the same as those of the SCD patients. Determination of hemoglobin genotypes of the study participants was based on hemoglobin electrophoresis. Information on demographic features of the study participants were collected using a structured questionnaire. A mid-steam urine sample was obtained from each of the study participants and analyzed in the Bacteriology Laboratory of the School of Biomedical and Allied Health Sciences of University of Ghana, which is located on the campus of KBTH.

### 2.2. Analysis of Urine Specimens

Urine specimens were aseptically inoculated onto plates of Blood agar, MacConkey agar, and Cysteine Lactose Electrolyte Deficient agar using a standard loop calibrated to hold 0.01 mL of urine. The plates were incubated at 37 °C aerobically for 18–24 h. After incubation bacterial colonies on the agar plates were counted and the results multiplied by the loop volume. A bacterial count of 1 × 10^5^ per mL was considered as significant bacteriuria, while counts less than 1 × 10^5^ per mL were considered as insignificant bacterial growth [1]. Bacterial isolates were identified based on colonial morphology, Gram stain, and a battery of biochemical tests [19].

### 2.3. Antibiotic Susceptibility Testing of Uropathogens

A modified form of the Kirby Bauer method was employed to evaluate the antibiotic susceptibility of bacteria isolated from urine specimens [20,21]. The antibiotics tested included co-trimoxazole, ceftizoxime, chloramphenicol, cephalexin, tetracycline, ciprofloxacin, amikacin, ampicillin, sparfloxacin, ofloxacin, norfloxacin, and levofloxacin (Oxoid Ltd., Basingstoke, UK). The antibiotic susceptibility testing procedure that was employed is briefly described as follows. Pure culture of the test organism was emulsified in peptone water until the turbidity was comparable with that of 0.5% McFarland’s standard solution. A loopful of the suspension of the test organism was transferred onto a Mueller–Hinton agar plate, and a sterile cotton swab was then used to streak the entire surface of the agar. Sterile forceps were used to apply the antibiotic discs to the surface of the agar plate and incubated at 37 °C for 18–24 h. Zone diameters formed around the antibiotic discs were measured and classified as sensitive or resistant based on the Clinical Laboratory Standard Institute (CLSI) break point system [21].

### 2.4. Data Analysis

Data were analyzed using STATA 11.0 (SPSS Inc., Chicago, IL, USA). First, descriptive analyses including computation of arithmetic means, frequencies, and percentages were carried out on the study variables. The study variables were compared between the SCD patients and control group using the Student’s *t*-test for continuous variables and the chi-square test for categorical variables. Univariable associations were performed between bacteriuria and demographic features of the SCD patients. Subsequently, variables significantly associated with bacteriuria were used as independent variables in a logistic regression analysis to identify determinants of bacteriuria. Significance of variables was assessed by *p*-values, odds ratios, and confidence intervals; *p*-values < 0.05 were interpreted as significant.

### 2.5. Ethics Statement

The study was approved by the by the Ethical and Protocol Review Committee of the School of Allied Health Sciences, University of Ghana, and informed consent was obtained from the study participants.

## 3. Results

Of the 110 SCD patients recruited in the study, 77 had the HbSS genotype and 33 the HbSC genotype. The demographic characteristics of the SCD patients and healthy controls are summarized in Table 1. The gender distribution of the SCD patients and healthy controls were the same, with 42 males and 68 females. The age distributions of the two groups of study subjects were similar: mean age of SCD patients was 26.0 (SD = 11.4) years, whereas that of healthy controls was 24.0 (SD = 12.0) years. The marital status of the SCD patients and healthy controls were also similar, with the majority being unmarried. The pattern of occupation of the SCD patients and controls were different; majority (50.9%) of the SCD patients were unemployed while among the control group, majority (70%) were professionals. There was no significant difference between the SCD patients and the controls in demographic features with the exception of occupation.

Prevalence of ASB and the associated causative organisms identified by culture among SCD patients and healthy controls are shown in Table 2. The overall ASB prevalence was significantly higher among SCD patients (17.2%, 19/110) than among the control group (8.2%, 9/110), and the relative risk was 2.11 (*p* = 0.0431; CI = 1.00–4.45). Prevalence of ASB was the same in male SCD patients and their healthy counterparts (2.4%). Prevalence of ASB was, however, higher among female SCD patients (26.5%) compared to their healthy counterparts (11.8%), though the difference was not significant (*p* = 0.054). Among both SCD patients and healthy controls, females had a higher prevalence of ASB compared to males; however, significant association of ASB and sex was observed only among the SCD patients (*p* = 0.001). In the logistic regression, being female emerged as a predictor of ASB among the SCD patients (OR = 14.76; CI = 11.23–18.29; *p* = 0.0103). Age, marital status, and occupation of the SCD patients did not show any significant association with ASB.

The most common organism isolated from both the SCD patients and healthy controls was coagulase-negative *Staphylococcus* spp., followed by *Escherichia coli*. Generally, causative organisms of bacteriuria did not show any significant differences between the SCD patients and the control group. However, organisms isolated from the SCD patients were more diverse and included *Enterobacter* spp., *Citrobacter* spp., *Klebsiella oxytoca,* and *Candida* spp., which were not isolated from the healthy controls. 

Antibiotic susceptibility patterns of bacteria isolated from urine specimens of the study participants are shown in Table 3. Generally, susceptibility patterns varied widely among isolates of the same organism. For *E. coli*, the main uropathogen, all the isolates were susceptible to amikacin, sparfloxacin, and norfloxacin, but were resistant to ampicillin.

## 4. Discussion

In this study, we investigated ASB among SCD patients for the first time in Ghana and observed a prevalence of 17%. By comparison, a similar study in Nigeria reported ASB prevalence of 4% [18], while another study in Jamaica reported a prevalence of 10.9% [22]. One particular strength of our study was the inclusion of a control group of people without SCD, which helped us to quantify the risk of ASB among people with SCD to be two-fold. Sickle cell disease patients have altered blood flow in the renal vasculature, which causes papillary necrosis and loss of urinary concentrating and acidifying ability of the nephrons [23,24,25]. This results in the formation of abnormally dilute and alkaline urine, which favors bacterial proliferation and partly explains the relatively higher prevalence of ASB among the SCD patients in this study [24].

Being female was found to be a determinant of ASB among the SCD patients, which is in keeping with previous studies among both SCD patients and healthy people [2,12,24]. Close proximity of the female urethral meatus to the anus, shorter urethra and sexual intercourse have been reported as factors accounting for the higher prevalence of urinary infections among females compared to males [2]. The relatively higher prevalence of urinary infections among females is also thought to be related to a relative deficiency in secretory IgA antibody response from the mucosal surface of the urogenital tract of females compared with males [26].

In line with other studies [27,28,29,30,31], coagulase-negative *Staphylococcus* and *E. coli* were the predominant organisms isolated from the urine specimens. Coagulase‑negative *Staphylococcus* spp. appears to be isolated more frequently from patients with ASB compared with those with symptomatic infection [27,28,29]. Thus, it is not surprising that coagulase‑negative *Staphylococcus* was the most predominant organism isolated from the ASB cases among both the SCD patients and controls. The wide diversity of causative organisms isolated from the SCD patients compared to the controls probably reflects the immune impairment in the former [9]. Interestingly, organisms that were isolated exclusively from the SCD patients (*Enterobacter* spp., *Citrobacter* spp., *Klebsiella oxytoca,* and *Candida* spp.) have been implicated in infections in people with diseases associated with immune suppression such as cancer and diabetes [28,32].

In the current study, *E. coli* resistance to ampicillin, which is a potential antibiotic for treatment of UTI, is worth noting. The high prevalence (100%) of urinary *E. coli* resistance to ampicillin in this study has been previously reported in Ghana [33]. Ampicillin has been on the Ghanaian market for a long time, and there is evidence of a high rate of misuse of this antibiotic [34]. Thus, it is not surprising to observe such high levels of ampicillin resistance. Our data on *E. coli* resistance to ampicillin represents one of the many examples where antibiotic misuse appears to be completely reversing the efficacy of antibiotics. This poses a major public health problem especially, in developing countries like Ghana where antibiotic treatment options are limited. It is important to note the challenges associated with the discovery of new antibiotics in recent times and thus, if the problem of antibiotic resistance is not curbed, the world may be approaching an era of completely failed antibiotics. Third-generation cephalosporins are relatively expensive and are therefore not subjected to frequent use in Ghana. This may partly explain the absence of *E. coli* resistance to several third generation cephalosporins in this study. It is important to control the use of these drugs so as to maintain their effectiveness for a longer period.

The findings of the study has important clinical implications for SCD patients who were found to have a two-fold risk of developing ASB compared to the healthy controls. In healthy people, ASB does not directly affect health and is considered a benign condition [35]. However, in untreated pregnant women with ASB, a significant proportion of them will eventually develop pyelonephritis, which may lead to delivery of a premature and/or low birth weight baby [6,8]. In SCD patients, ASB may indeed lead to renal damage due to several anatomical and physiological abnormalities [13,24,25]. Our study was a cross-sectional study, and we were therefore not able to evaluate if the ASB cases among the SCD patients actually progressed to symptomatic UTI. A study in Jamaica showed a significant association between ASB and symptomatic UTI [23]. In SCD patients, symptomatic UTI is associated with bacteraemia, pneumonia, and osteomyelitis [36]. Further studies are needed to confirm that ASB in SCD patients really leads to symptomatic UTI. Should this be the case, then the high risk of ASB among SCD patients as illustrated by this study indicates that early screening and treatment for ASB among SCD patients may be necessary. This is particularly important in resource-poor settings where renal replacement therapy is limited, and therefore early detection and management of ASB in SCD patients is crucial to prevent problems with kidney damage.

The main limitation of this study is that we were unable to follow up the SCD patients to determine outcome of ASB as symptomatic UTI or not.

## 5. Conclusions

Sickle cell disease patients in Ghana have a two-fold risk of developing ASB compared to healthy people, and the risk is particularly high among female SCD patients. The microbial etiology of ASB in the SCD patients appears to be more diverse compared to healthy people. Ampicillin is not suitable for empirical treatment of urinary infections in Ghana due to resistance of *E. coli* to this antibiotic.

## Figures and Tables

**Table 1:** Demographic features of the study participants.

Variable	Sickle Cell Disease Group	Control Group	Significance
	n (%)	n (%)	
Gender			*p* > 0.05
Male	42 (38.1)	42 (38.1)	
Female	68 (61.9)	68 (61.9)	
Mean age (years)	26.0 (SD = 11.4)	24.0 (SD = 12.0)	*p* > 0.05
Occupation			*p* < 0.05
Unemployed	56 (50.9)	9 (8.2)	
Artisans	40 (36.4)	24 (21.8)	
Professionals	14 (12.7)	77 (70)	
Marital status			*p* > 0.05
Married	23 (20.9)	26 (23.6)	
Single	85 (77.2)	82 (74.6)	
Widowed	2 (1.9)	2 (1.8)	

“n”—number or frequency.

**Table 2:** ASB prevalence and causative organisms of SCD patients and healthy controls.

Parameter	SCD Patients		Control Group	
	n	%	n	%
Overall ASB prevalence	19	17.3	9	8.2
ASB prevalence in males	1	2.4	1	2.4
ASB prevalence in females	18	26.5	8	11.8
Causative organisms				
Coagulase negative *Staphylococcus* spp.	4	3.6	5	4.5
*Escherichia coli*	4	3.6	2	1.8
*Enterobacter* spp.	3	2.7	0	0
*Streptococcus* spp.	3	2.7	1	0.9
*Staphylococcus aureus*	1	0.9	1	0.9
*Klebsiella oxytoca*	1	0.9	0	0
*Citrobacter* spp.	1	0.9	0	0
*Candida* spp.	1	0.9	0	0

“n”—number or frequency; SCD—sickle cell disease; ASB—asymptomatic bacteriuria. Parameters showing statistical significance were: difference in overall ASB prevalence of SCD patients and control group (*p* = 0.043); difference in ASB prevalence of SCD male and female patients (*p* = 0.001).

**Table 3:** Antibiogram of urinary bacterial isolates from SCD patients and healthy controls.

ISOLATES	SOURCE	BA	CL	CH	PR	TE	CP	AK	AS	SC	OF	NX	LE
*E. coli*	SCD	S	S	R	S	S	S	S	R	S	S	S	S
*E. coli*	SCD	S	S	R	R	R	S	S	R	S	S	S	S
*E. coli*	SCD	R	S	S	S	R	S	S	R	S	S	S	S
*E. coli*	SCD	S	R	S	R	R	R	S	R	S	S	S	S
*E. coli*	NSCD	S	S	S	S	R	S	S	R	S	S	S	S
*E. coli*	NSCD	R	R	R	R	R	S	S	R	S	R	S	R
CNS	NSCD	R	S	S	S	S	S	S	S	S	S	S	S
CNS	NSCD	R	R	R	S	S	S	S	R	S	S	S	S
CNS	NSCD	R	I	R	S	S	S	S	R	S	S	S	S
CNS	NSCD	S	R	R	S	R	S	S	R	S	S	S	S
CNS	NSCD	R	S	S	S	R	S	S	R	S	S	S	S
CNS	SCD	R	R	S	S	R	R	S	S	I	S	S	S
CNS	SCD	S	S	S	S	S	S	S	S	S	S	S	S
CNS	SCD	S	R	S	R	R	S	I	R	S	S	S	S
CNS	SCD	S	S	R	S	S	S	S	R	S	S	S	S
*K. oxytoca*	SCD	R	S	R	R	R	S	S	R	S	S	S	S
*S. aureus*	NSCD	S	S	S	S	S	S	S	R	S	S	S	S
*S. aureus*	SCD	S	S	S	S	S	S	S	R	S	S	S	S
*Enterobacter* sp.	SCD	S	S	R	S	S	S	S	S	S	S	S	S
*Enterobacter* sp.	SCD	S	S	R	R	R	S	S	R	S	S	S	S
*Enterobacter* sp.	SCD	R	S	R	S	S	S	S	R	S	S	S	S
*Citrobacter* sp.	SCD	R	R	S	R	R	S	R	R	S	S	S	S

*S. aureus*—*Staphylococcus aureus*; *K. oxytoca*—*Klebsiella oxytoca*; CNS—coagulase-negative *Staphylococcus* sp.; *E. coli*—*Escherichia coli*; SCD—sickle cell disease; NSCD—non-sickle cell disease; R—resistant; S—susceptible; I—intermediate; BA—cotrimoxazole; AK—amikacin; CL—ceftizoxime; AS—ampicillin; CH—chloramphenicol; SC—sparfloxacin; PR—cephalexin; OF—ofloxacin; TE—tetracycline; NX—norfloxacin; CP—ciprofloxacin; LE—levofloxacin.
